# Time‐ and Region‐Specific Effects of Intranasal Insulin on Oxidative Stress Parameters in the Rat Brain

**DOI:** 10.1096/fj.202601712R

**Published:** 2026-07-05

**Authors:** Jelena Osmanovic Barilar, Leonarda Vlahov, Antonia Krsnik, Luka Mihalic, Ana Babic Perhoc, Davor Virag, Jan Homolak, Melita Salkovic‐Petrisic, Ana Knezovic

**Affiliations:** ^1^ Department of Pharmacology, School of Medicine University of Zagreb Zagreb Croatia; ^2^ Croatian Institute for Brain Research, School of Medicine University of Zagreb Zagreb Croatia; ^3^ Interfaculty Institute for Microbiology and Infection Medicine Tübingen University of Tübingen Tübingen Germany; ^4^ Cluster of Excellence EXC 2124 Controlling Microbes to Fight Infections University of Tübingen Tübingen Germany; ^5^ M3‐Research Center for Malignome, Metabolome and Microbiome University of Tübingen Tübingen Germany

**Keywords:** brain, insulin, intranasal administration, oxidative stress, redox homeostasis

## Abstract

Understanding how intranasal insulin affects brain signaling and metabolism is essential for elucidating its therapeutic potential in neurodegenerative disorders with underlying metabolic dysfunction, such as Alzheimer's disease (AD). Oxidative stress, which increases with aging, has been observed in both AD and Type 2 diabetes, indicating a potential link between oxidative stress, brain insulin resistance and cognitive impairment. This study examined how intranasal insulin affects redox homeostasis across different brain regions and time points. Male Wistar rats received 2 IU of insulin intranasally and were sacrificed 3, 7.5, 15, 30, 60, and 120 min post‐administration. Six animals served as intact controls. Redox homeostasis was assessed by measuring lipid peroxidation, total reductive capacity, thiol concentrations, and superoxide dismutase activity in plasma, nasal epithelia, and brain regions. The results were correlated with insulin signaling markers. Intranasal insulin induced rapid but regionally diverse redox responses. The most pronounced alterations occurred in nasal epithelia, where respiratory and olfactory regions exhibited distinct and opposing patterns. In the brain, significant alterations, particularly in thiol‐related parameters, were observed across multiple regions including cortices, hippocampus, hypothalamus, olfactory bulb, and cerebellum. Plasma redox parameters remained largely unchanged, supporting the predominantly central action of intranasally delivered insulin. Correlation analyses revealed associations between oxidative stress markers and insulin signaling parameters, suggesting complex interactions between metabolic signaling pathways and redox regulation. These findings demonstrate that intranasal insulin modulates redox homeostasis in a rapid, region‐specific, and time‐dependent manner, highlighting the importance of spatial and temporal factors in insulin‐mediated regulation of brain oxidative balance.

## Graphical abstract

Intranasal insulin induced time‐dependent heterogeneous oxidative stress–related responses across brain regions, with opposite patterns in the olfactory and respiratory epithelia, while plasma redox parameters were minimally affected. These redox modulations were found associated with insulin signaling markers, including C‐peptide, AMPK activity, and IRS inhibition. The findings demonstrate a dynamic interaction between insulin signaling and redox regulation following intranasal insulin administration.

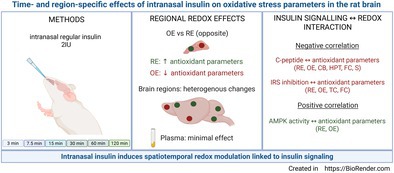

## 1 Introduction

Although insulin has historically been viewed primarily as a peripheral hormone regulating glucose homeostasis, accumulating evidence demonstrates that insulin also exerts important physiological effects within the central nervous system. Insulin receptors are widely distributed throughout the brain, particularly in regions involved in cognition and metabolic regulation, such as the hypothalamus, hippocampus, cortex, olfactory bulb, and cerebellum. These observations, together with evidence suggesting that small amounts of insulin may be synthesized locally in the brain, support the concept that insulin plays a significant neuromodulatory role. Nevertheless, most insulin present in the brain is believed to originate from the peripheral circulation and enters the central nervous system via receptor‐mediated transport across the blood–brain barrier (BBB) [1, 2, 3]. In the brain, insulin signaling participates in numerous processes essential for neuronal function and survival. These include regulation of neuronal growth, dendritic outgrowth, synaptic density, structural plasticity, and structural maintenance [4, 5]. Insulin also contributes to neuronal plasticity and learning‐related processes by influencing signaling pathways such as PI3K/Akt and MAPK, which regulate cellular metabolism and survival [2, 6, 7]. Because of these diverse roles, efficient brain insulin signaling is considered a critical factor in maintaining cognitive performance and healthy brain aging. Conversely, impaired insulin signaling in the brain has been increasingly implicated in the development of neurodegenerative disorders, particularly Alzheimer's disease (AD) [7]. Neuroimaging studies have revealed that reductions in cerebral glucose metabolism may occur more than a decade before the onset of clinical dementia symptoms [8]. These findings have led to the hypothesis that metabolic dysfunction and insulin resistance within the brain contribute substantially to AD pathogenesis. Brain insulin resistance is characterized by reduced insulin availability in the central nervous system or decreased responsiveness of neuronal cells to insulin signaling [9, 10]. As a result, neuronal glucose utilization becomes impaired, leading to metabolic stress, disrupted cellular signaling, and increased vulnerability to neurodegeneration. Given the growing recognition of the role of insulin signaling in brain physiology and pathology, therapeutic strategies aimed at restoring central insulin activity have attracted considerable attention. One particularly promising approach is the intranasal administration of insulin, which allows direct delivery of the hormone to the brain while minimizing systemic metabolic effects such as hypoglycemia [10, 11]. Intranasal delivery exploits anatomical connections between the nasal cavity and the brain through olfactory and trigeminal pathways, enabling rapid transport of peptides to the central nervous system and bypassing the BBB [12]. Clinical studies have demonstrated that intranasal insulin can improve memory performance, cognitive function, and cerebral metabolic activity in individuals with mild cognitive impairment and AD. However, not all studies have reported consistent benefits, suggesting that treatment outcomes may depend on factors such as disease stage, insulin formulation, sex, dosage, treatment time, or genetic background [13, 14]. In addition to its effects on cognition, insulin also plays an important role in mitochondrial function and oxidative stress regulation in the brain [15]. Recent studies demonstrate a strong association between insulin resistance and an increased risk of neurodegeneration, dementia, depression, and mild cognitive impairment. Although these conditions may partly result from metabolic disturbances such as hyperglycemia, hyperinsulinemia, dyslipidemia, and chronic inflammation, growing evidence highlights mitochondrial dysfunction and oxidative stress as central mechanisms. The brain is particularly vulnerable to oxidative damage due to its high oxygen consumption, abundance of polyunsaturated lipids, relatively low antioxidant capacity, and high levels of redox‐active metal ions, making reactive oxygen species (ROS) overproduction especially detrimental to neuronal function and survival [16]. Understanding how insulin signaling interacts with mitochondrial function and oxidative stress in the central nervous system may therefore provide valuable insights into the mechanisms underlying neurodegenerative diseases and support the development of novel therapeutic approaches aimed at restoring brain metabolic homeostasis. The aim of this study was to investigate the effects of intranasal insulin administration on redox homeostasis in the brain and nasal epithelia, focusing on the temporal dynamics of oxidative stress markers in relation to insulin signaling.

## 2 Materials and Methods

### 2.1 Animals and Ethical Approval

Experiments were conducted on male Wistar rats (4‐months‐old), with a body mass of 300–400 g, bred at the Croatian Institute for Brain Research, School of Medicine, University of Zagreb. All rats were housed in cages (2–3 rats per cage) under controlled environmental conditions (22°C ± 2°C, 12 h light/dark cycle) in an authorized unit of the Institute, fed standard chow and water ad libitum. All experimental procedures were conducted in accordance with the European Directive 2010/63/EU for the protection of animals used for scientific purposes and were approved by the appropriate institutional ethics committee, the Ministry of Agriculture of the Republic of Croatia (license number EP 394/2023). Animals were handled exclusively by officially trained personnel.

### 2.2 Experimental Design and Intranasal Insulin Administration

Thirty‐six healthy 4‐month‐old rats received a single dose of 2 IU of human regular insulin intranasally (10 μL/1 IU per nostril) [17]. Intranasal administration was performed while the animals were awake, without anesthesia. The insulin solution was administered slowly using a pipette, in several drops, to a total volume of 20 μL. The animal was kept in this position for 5–10 s to allow spontaneous inhalation of the applied insulin solution. No bleeding or coughing was observed during the procedure. The dose used in this study was selected based on previous studies demonstrating beneficial effects on cognitive functions in rats and mice [18, 19, 20, 21, 22, 23, 24]. Animals were sacrificed at different time points after intranasal insulin administration (6 animals per group): 3 min, 7.5 min, 15 min, 30 min, 1 h, and 2 h. Additionally, 6 animals served as an untreated (intact) group.

### 2.3 Tissue Collection and Sample Preparation

The animals were sacrificed under deep general anesthesia with thiopental and diazepam (70 mg/kg and 7 mg/kg, intraperitoneally, i.p.). Blood was collected from the retro‐orbital sinus into heparinized microtubes and centrifuged for 10 min at 900 × g at 4°C. The supernatant (plasma) was collected and stored at −80°C. All animals were decapitated, after which the brains and nasal epithelia were rapidly removed, and the respiratory epithelium (RE), olfactory epithelium (OE), brainstem (BS), cerebellum (CB), olfactory bulb (OFB), striatum (S), hippocampus (HPC), hypothalamus (HPT), frontal (FC), parietal (PC), and temporal (TC) cortex were isolated. All listed tissues were then snap‐frozen in liquid nitrogen and stored at −80°C. Tissue samples for analysis were thawed and homogenized in five volumes of lysis buffer containing 1 M Tris (pH 8.0), 1 M NaCl, 0.005 M EDTA, 1 M DTT, 0.01 M sodium vanadate, and protease and phosphatase inhibitors. The prepared homogenates were centrifuged at 16060 × g for 10 min at 4°C. The resulting supernatants were collected and stored at −80°C until further use. Protein concentration was determined using the Lowry protein assay.

### 2.4 Indirect Assessment of Lipid Peroxidation by Quantification of Thiobarbituric Acid Reactive Substances (TBARS)

Supernatants of tissue homogenates (20 μL) were supplemented with 70 μL of ddH_2_O to increase the reaction volume. The samples were then mixed with 120 μL of TBA–TCA reagent (0.4% thiobarbituric acid dissolved in a 15% trichloroacetic acid solution). Standards prepared in advance (1 μM, 10 μM, 25 μM, 50 μM, and 100 μM malondialdehyde (MDA) tetrabutylammonium salt) were processed in the same manner. The samples were vortexed, centrifuged, and then placed in a heating block at 95°C. The reaction was monitored by observing color development in the samples. After completion of the reaction (lasting 15–20 min, depending on the sample), the samples were removed from the heater and centrifuged again to allow condensation to collect at the bottom of the tube. The resulting colored adduct of thiobarbituric acid and reactive aldehydes was extracted by adding n‐butanol in a volume equal to the final volume of each sample, followed by vortexing. After the addition of butanol and vortexing, the samples were left for several minutes to allow complete phase separation. The absorbance of the butanol fraction (100 μL) was measured at a wavelength of 540 nm using Infinite F200 PRO multimode microplate reader (Tecan, Männedorf, Switzerland) [25]. TBARS concentration was calculated based on a calibration curve generated from MDA standard samples. Results are presented as concentration per μg of protein in the sample.

### 2.5 Determination of Free Thiol Group Concentration and Low‐Molecular‐Weight Thiol Concentration

The concentrations of free thiol groups (SH; sulfhydryls) and low‐molecular‐weight thiols (LMWT) were determined based on quantification of the formation of 5‐thio‐2‐nitrobenzoic acid (TNB) in the reaction of sulfhydryls with 5,5′‐dithiobis(2‐nitrobenzoic acid) (DTNB) [26]. Twenty‐five microliters of homogenate supernatant were mixed with a 4% sulphosalicylic acid (SSA) solution at a 1:1 volume ratio and incubated on ice for 60 min. After incubation, the tubes were centrifuged for 10 min at 10000 × g, and 30 μL of the supernatant were transferred into separate microtubes. The remaining supernatant was discarded by inverting the original tubes onto a paper towel, leaving only the pellet. Subsequently, 35 μL of DTNB solution (4 mg/mL in 5% sodium citrate solution) were added to both the tubes containing supernatant and those containing the pellet, with careful resuspension of the pellet. The samples were incubated for 10 min, after which the contents of all tubes were transferred to plates and absorbance was measured at 405 nm using an Infinite F200 PRO multimode microplate reader (Tecan, Männedorf, Switzerland). LMWT and SH concentrations were calculated using a molar extinction coefficient of 14 150 M^−1^·cm^−1^. Thiol concentrations were normalized to protein content, except in plasma.

### 2.6 Determination of Total Reductive Capacity

To assess overall redox homeostasis, total reductive capacity was measured using the decolorization of the metastable radical cation 2,2′‐azino‐bis(3‐ethylbenzothiazoline‐6‐sulfonic acid) (ABTS) [27]. The metastable ABTS radical cation was generated by mixing 7 mM ABTS with 2.45 mM K_2_S_2_O₈. The resulting solution was incubated overnight in the dark at room temperature. On the following day, the working solution was prepared by diluting the ABTS radical cation solution at a 1:40 ratio. One microliter of each sample was mixed with 100 μL of the working solution of ABTS radical cation. Controls contained only the ABTS radical cation solution without sample. Total reductive capacity was calculated based on the difference in absorbance between the non‐reduced ABTS radical cation (control wells) and the absorbance measured in wells containing samples. Absorbance was measured at 405 nm using an Infinite F200 PRO multimode microplate reader. Results were normalized to protein content.

### 2.7 Determination of Superoxide Dismutase Activity

Superoxide dismutase (SOD) activity was determined indirectly by measuring the degree of auto‐oxidation of 1,2,3‐trihydroxybenzene (THB; pyrogallol) [28]. The THB solution was prepared by dissolving 60 mM THB in 1 mM HCl. The working buffer for SOD activity measurement contained 0.05 M Tris–HCl and 1 mM Na_2_EDTA in ddH_2_O, with the final pH adjusted to 8.2. For each measurement, 10 μL of homogenate samples from brain regions and epithelia were added to the microplate wells. The reaction solution was prepared by mixing 80 μL of THB with 4000 μL of working buffer, and 100 μL of this solution were added to each well. Immediately thereafter, absorbance was measured at 450 nm every 60 s for a total period of 300 s. Auto‐oxidation of THB generates superoxide radicals, and SOD activity is inversely proportional to the increase in absorbance—higher enzyme activity results in a slower increase in signal. During the first 300 s, the reaction generally follows a linear course with a constant slope. In addition, the activity of the mitochondrial form of SOD (Fe/Mn‐SOD) was assessed, while cytosolic Cu/Zn‐SOD activity was inhibited using 2 mM potassium cyanide (KCN) [29], enabling selective measurement of mitochondrial Mn‐SOD activity. SOD activity results are presented as the difference in THB absorbance between 4 and 1 min and normalized to protein content.

### 2.8 Statistical Analysis

Results are presented as box‐and‐whisker plots, and differences between groups were analyzed using the Kruskal–Wallis analysis of variance. The multiple‐comparisons test used was the uncorrected Dunn's test, with the alpha value set at 0.05. All analyses were performed using GraphPad Prism 8. In addition, the obtained results were correlated with previously published results from the same tissue samples [17] of insulin signaling analysis, including glucose, insulin, and C‐peptide concentrations, as well as insulin receptor substrate activity (p/tIRS608 and p/tIRS307), and AMP‐activated kinase activity (p/tAMPK). For individual brain regions, epithelia, and plasma, Spearman's rank correlation coefficient (ρ) was calculated for the measured variables. Correlations are presented as heatmaps using the JASP software. Correlation analyses were restricted to samples obtained from insulin‐treated groups. Control animals were not included, as the control groups used for oxidative stress measurements were not identical to those used in the insulin signaling analyses. To maintain comparability between datasets, correlations between oxidative stress parameters and insulin signaling markers were performed exclusively on matched samples from insulin‐treated animals.

## 3 Results

### 3.1 Plasma Redox Effects of Intranasal Insulin

**FIGURE 1:**
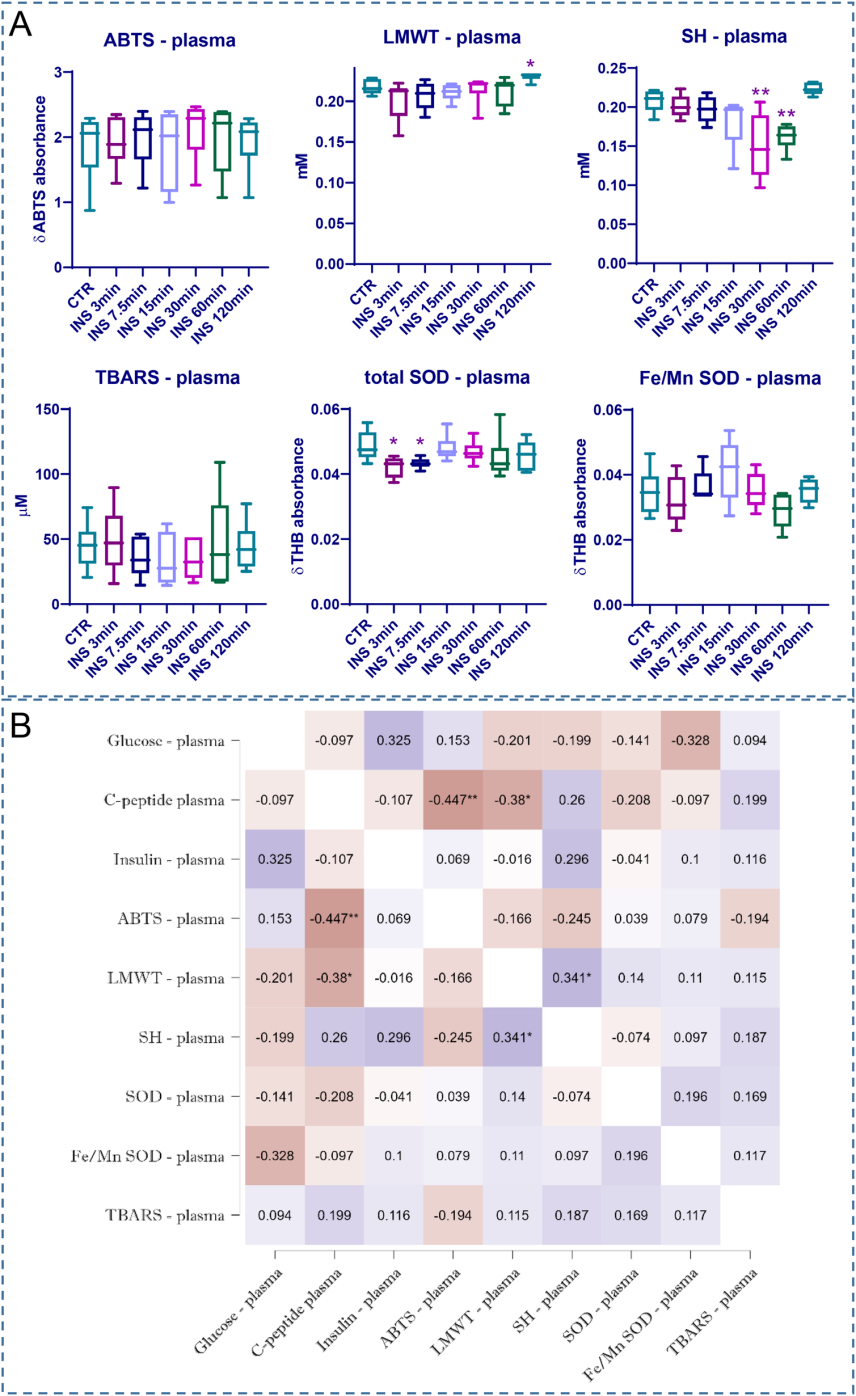
Time‐dependent effect of insulin on redox homeostasis in plasma. (A) Free thiol group (SH) concentration, low‐molecular‐weight thiol (LMWT) concentration, total reducing capacity (ABTS), superoxide dismutase activity (total SOD), Fe/Mn superoxide dismutase activity (Fe/Mn SOD), and indirect assessment of lipid peroxidation by quantification of thiobarbituric acid reactive substances (TBARS) were determined in plasma. Results are presented as box‐and‐whisker plots, and differences between groups were analyzed using Kruskal–Wallis analysis of variance followed by an uncorrected Dunn's test, with the *p* value set at 0.05 (*p < 0.05 vs. control, **p < 0.01 vs. control, ***p < 0.001 vs. control). (B) Redox homeostasis parameters were correlated with insulin, C‐peptide and glucose concentrations. Spearman's rank correlations are presented as a heatmap, with statistically significant correlations indicated as *p < 0.05, **p < 0.01, ***p < 0.001.

LMWT concentration was increased 120 min after intranasal administration (+6.1%), whereas SH concentration was decreased 30 (−28.4%) and 60 min (−22.5%) after administration compared with the control group. In addition, insulin acutely reduced total SOD activity in plasma at 3 (−13.2%) and 7.5 min (−10.9%) after its administration. No changes were observed in total reducing capacity (ABTS), TBARS levels, or Fe/Mn SOD activity in plasma after intranasal insulin administration compared with control (Figure 1A). No strong correlations were observed between oxidative stress parameters and insulin signaling markers in plasma (Figure 1B).

### 3.2 Redox Modulation in Nasal Epithelia

**FIGURE 2:**
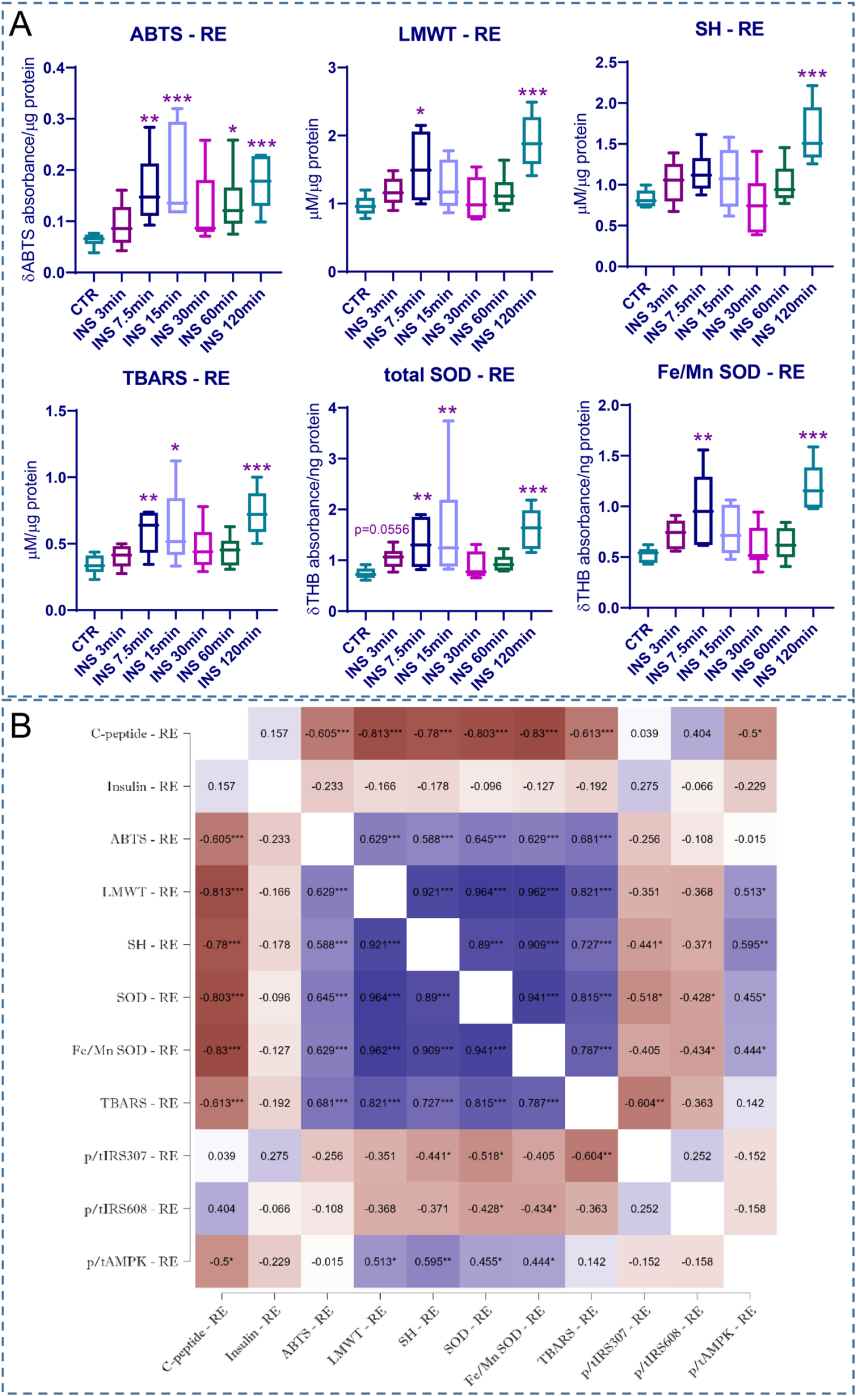
Time‐dependent effect of insulin on redox homeostasis in respiratory epithelium. (A) Free thiol group (SH) concentration, low‐molecular‐weight thiol (LMWT) concentration, total reducing capacity (ABTS), superoxide dismutase activity (total SOD), Fe/Mn superoxide dismutase activity (Fe/Mn SOD), and indirect assessment of lipid peroxidation by quantification of thiobarbituric acid reactive substances (TBARS) were determined in respiratory epithelium (RE). Results are presented as box‐and‐whisker plots, and differences between groups were analyzed using Kruskal–Wallis analysis of variance followed by an uncorrected Dunn's test, with the *p* value set at 0.05 (*p < 0.05 vs. control, **p < 0.01 vs. control, ***p < 0.001 vs. control). (B) Redox homeostasis parameters were correlated with insulin and C‐peptide concentrations, as well as with measures of insulin receptor substrate activation (p/tIRS608), insulin receptor substrate inhibition (p/tIRS307), and AMP‐activated kinase activation (p/tAMPK). Spearman's rank correlations are presented as a heatmap, with statistically significant correlations indicated as *p < 0.05, **p < 0.01, ***p < 0.001.

**FIGURE 3:**
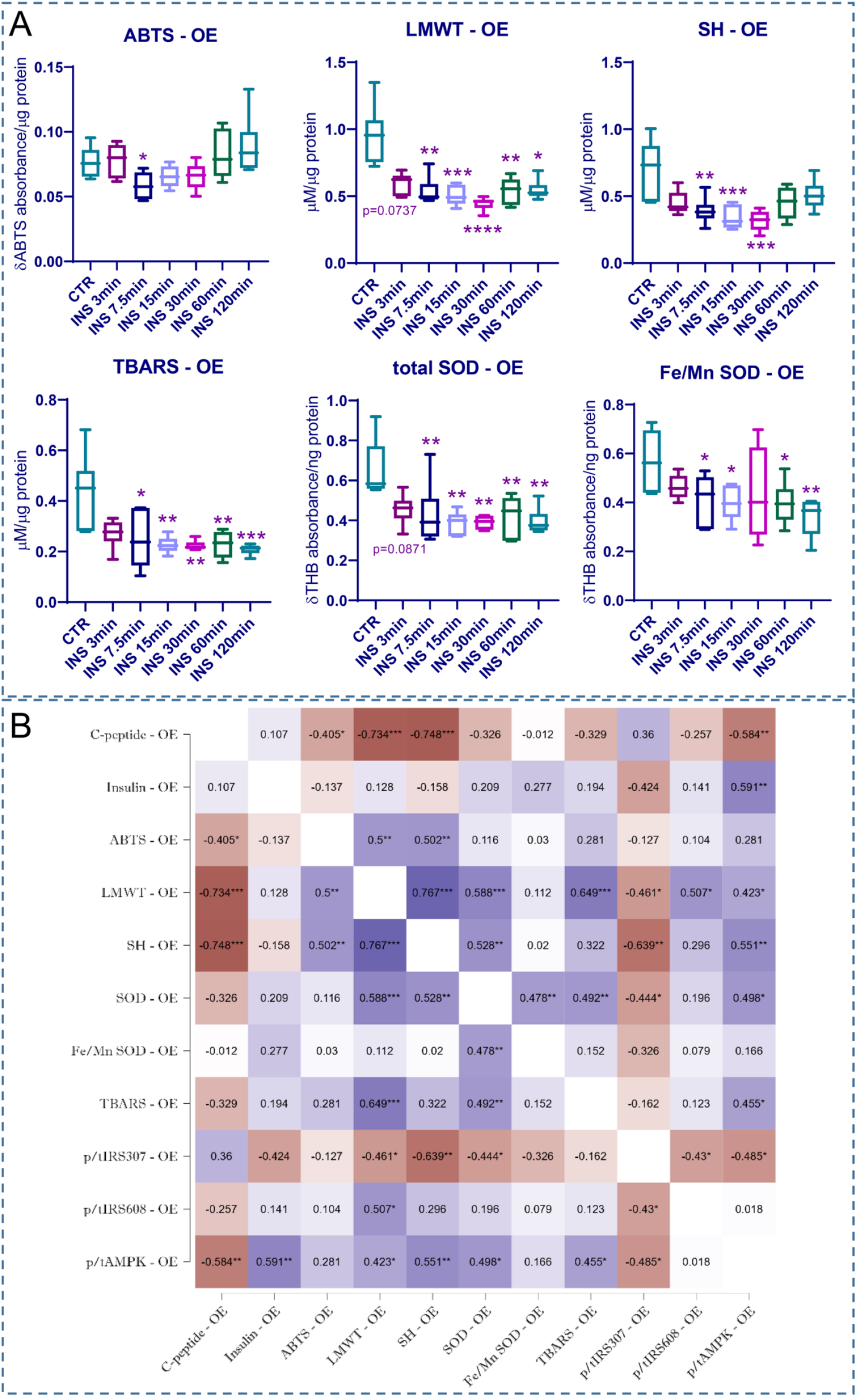
Time‐dependent effect of insulin on redox homeostasis in olfactory epithelium. (A) Free thiol group (SH) concentration, low‐molecular‐weight thiol (LMWT) concentration, total reducing capacity (ABTS), superoxide dismutase activity (total SOD), Fe/Mn superoxide dismutase activity (Fe/Mn SOD), and indirect assessment of lipid peroxidation by quantification of thiobarbituric acid reactive substances (TBARS) were determined in olfactory epithelium (OE). Results are presented as box‐and‐whisker plots, and differences between groups were analyzed using Kruskal–Wallis analysis of variance followed by an uncorrected Dunn's test, with the *p* value set at 0.05 (*p < 0.05 vs. control, **p < 0.01 vs. control, ***p < 0.001 vs. control). (B) Redox homeostasis parameters were correlated with insulin and C‐peptide concentrations, as well as with measures of insulin receptor substrate activation (p/tIRS608), insulin receptor substrate inhibition (p/tIRS307), and AMP‐activated kinase activation (p/tAMPK). Spearman's rank correlations are presented as a heatmap, with statistically significant correlations indicated as *p < 0.05, **p < 0.01, ***p < 0.001.

In nasal epithelia, intranasal insulin induced rapid and pronounced alterations in redox homeostasis. Total reductive capacity was significantly increased in the RE starting at 7.5 min after administration (+113% to 190%), while no marked changes in total reductive capacity were observed in the OE. Other oxidative stress–related parameters showed opposing trends between the two epithelia, being predominantly increased in the RE and decreased in the OE. In the OE, insulin significantly reduced LMWT concentrations at all time points, with reductions ranging from 38% to 53%. In contrast, LMWT levels in the RE were significantly increased at 7.5 (+58.4%) and 120 (+97.5%) minutes following intranasal administration. SH levels in the OE were significantly decreased at 7.5 (−44.41%), 15 (−51.69%), and 30 (−54.9%) minutes after insulin administration, whereas a significant increase in SH concentration was observed in the RE at 120 min (+94.75%). In the RE, TBARS (+73.8% and +115.3%, respectively) levels, as well as total SOD (+80.75% and +119.7%, respectively) and Fe/Mn SOD (+87.2% and +128%, respectively) activities, were increased at 7.5 and 120 min after insulin administration, while in the OE these parameters were decreased starting from 7.5 min following insulin administration. Spearman's rank correlation analysis was performed to examine correlations between the measured parameters and insulin signaling. In OE, there was a strong (*p* < 0.001) negative correlation between changes in C‐peptide concentration and LMWT (ρ = −0.734), as well as between C‐peptide and SH (ρ = −0.748) following insulin administration (Figure 3B). All measured parameters of oxidative stress were found negatively correlated with changes in C‐peptide concentration in RE (ρ = −605 to −830) (Figure 2B). Additionally, in both regions a significant (*p* < 0.01) positive correlation was observed between SH and p/tAMPK (ρ = +0.551 in OE, ρ = +0.595 in RE). Furthermore, a significant negative correlation between SH and p/tIRS307 was observed in the OE (ρ = −0.639) and between TBARS and p/tIRS307 in RE (ρ = −0.604) (Figures 2B and 3B).

### 3.3 Region‐Specific Effect of Insulin on Redox Homeostasis in the Brain

#### 3.3.1 Hippocampus

**FIGURE 4:**
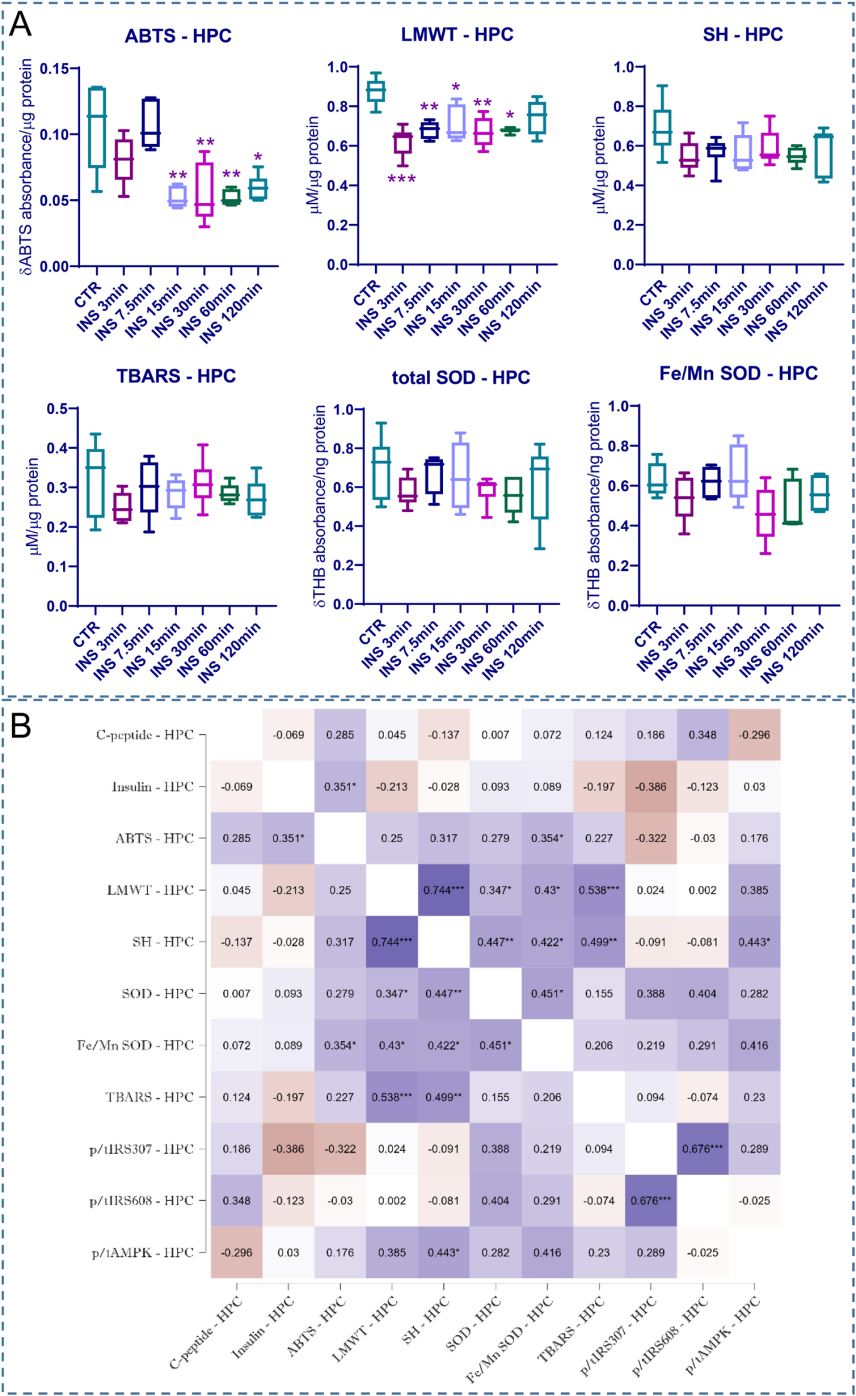
Time‐dependent effect of insulin on redox homeostasis in hippocampus. (A) Free thiol group (SH) concentration, low‐molecular‐weight thiol (LMWT) concentration, total reducing capacity (ABTS), superoxide dismutase activity (total SOD), Fe/Mn superoxide dismutase activity (Fe/Mn SOD), and indirect assessment of lipid peroxidation by quantification of thiobarbituric acid reactive substances (TBARS) were determined in hippocampus (HPC). Results are presented as box‐and‐whisker plots, and differences between groups were analyzed using Kruskal–Wallis analysis of variance followed by an uncorrected Dunn's test, with the *p* value set at 0.05 (*p < 0.05 vs. control, **p < 0.01 vs. control, ***p < 0.001 vs. control). (B) Redox homeostasis parameters were correlated with insulin and C‐peptide concentrations, as well as with measures of insulin receptor substrate activation (p/tIRS608), insulin receptor substrate inhibition (p/tIRS307), and AMP‐activated kinase activation (p/tAMPK). Spearman's rank correlations are presented as a heatmap, with statistically significant correlations indicated as *p < 0.05, **p < 0.01, ***p < 0.001.

Intranasal insulin administration did not induce significant changes in TBARS levels, SH concentrations, or total SOD activity in the HPC. A significant decrease in total reductive capacity was observed from 15 min and persisted until 120 min after administration, ranging from −44% to −51%. LMWT concentration was also significantly reduced at nearly all time points following intranasal insulin administration—3 (−28.95%), 7.5 (−22.41%), 15 (−19.44%), 30 (−23.67%), and 60 (−22.77%) minutes (Figure 4A). In the HPC, no significant association (ρ < 0.5 and *p* > 0.01) was observed between the measured oxidative stress parameters and insulin signaling parameters over time following intranasal insulin administration (Figure 4B).

#### 3.3.2 Hypothalamus

**FIGURE 5:**
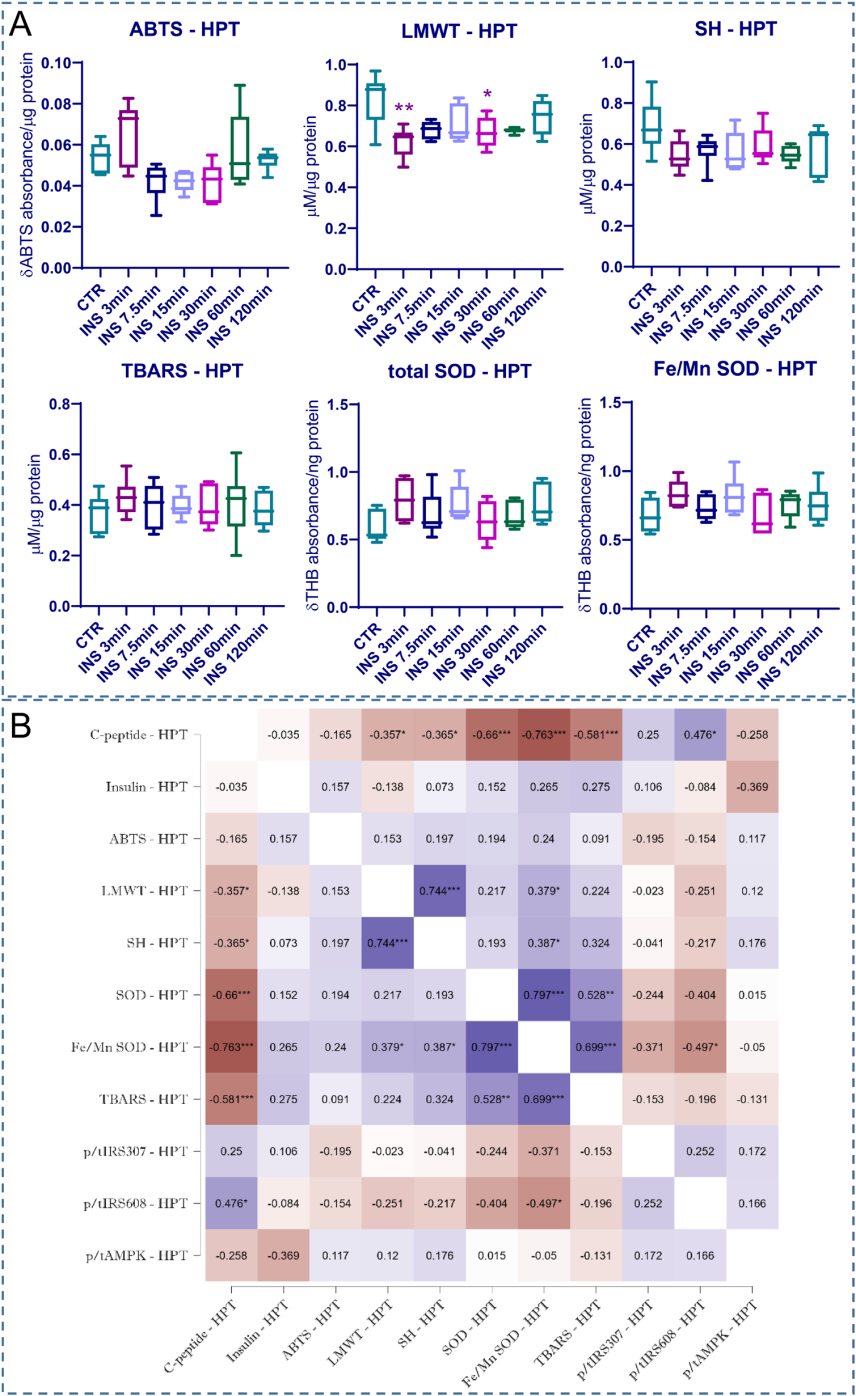
Time‐dependent effect of insulin on redox homeostasis in hypothalamus. (A) Free thiol group (SH) concentration, low‐molecular‐weight thiol (LMWT) concentration, total reducing capacity (ABTS), superoxide dismutase activity (total SOD), Fe/Mn superoxide dismutase activity (Fe/Mn SOD), and indirect assessment of lipid peroxidation by quantification of thiobarbituric acid reactive substances (TBARS) were determined in hypothalamus (HPT). Results are presented as box‐and‐whisker plots, and differences between groups were analyzed using Kruskal–Wallis analysis of variance followed by an uncorrected Dunn's test, with the *p* value set at 0.05 (*p < 0.05 vs. control, **p < 0.01 vs. control, ***p < 0.001 vs. control). (B) Redox homeostasis parameters were correlated with insulin and C‐peptide concentrations, as well as with measures of insulin receptor substrate activation (p/tIRS608), insulin receptor substrate inhibition (p/tIRS307), and AMP‐activated kinase activation (p/tAMPK). Spearman's rank correlations are presented as a heatmap, with statistically significant correlations indicated as *p < 0.05, **p < 0.01, ***p < 0.001.

Intranasal insulin reduced LMWT concentration at 3 (−25%) and 30 (−19.6%) minutes in the HPT (Figure 5A). In addition, a strong negative correlation was observed between C‐peptide concentration and redox parameters, including total SOD activity (ρ = −0.66), Fe/Mn SOD activity (ρ = −0.763), and TBARS concentration (ρ = −0.581) (Figure 5B).

#### 3.3.3 Olfactory Bulb

**FIGURE 6:**
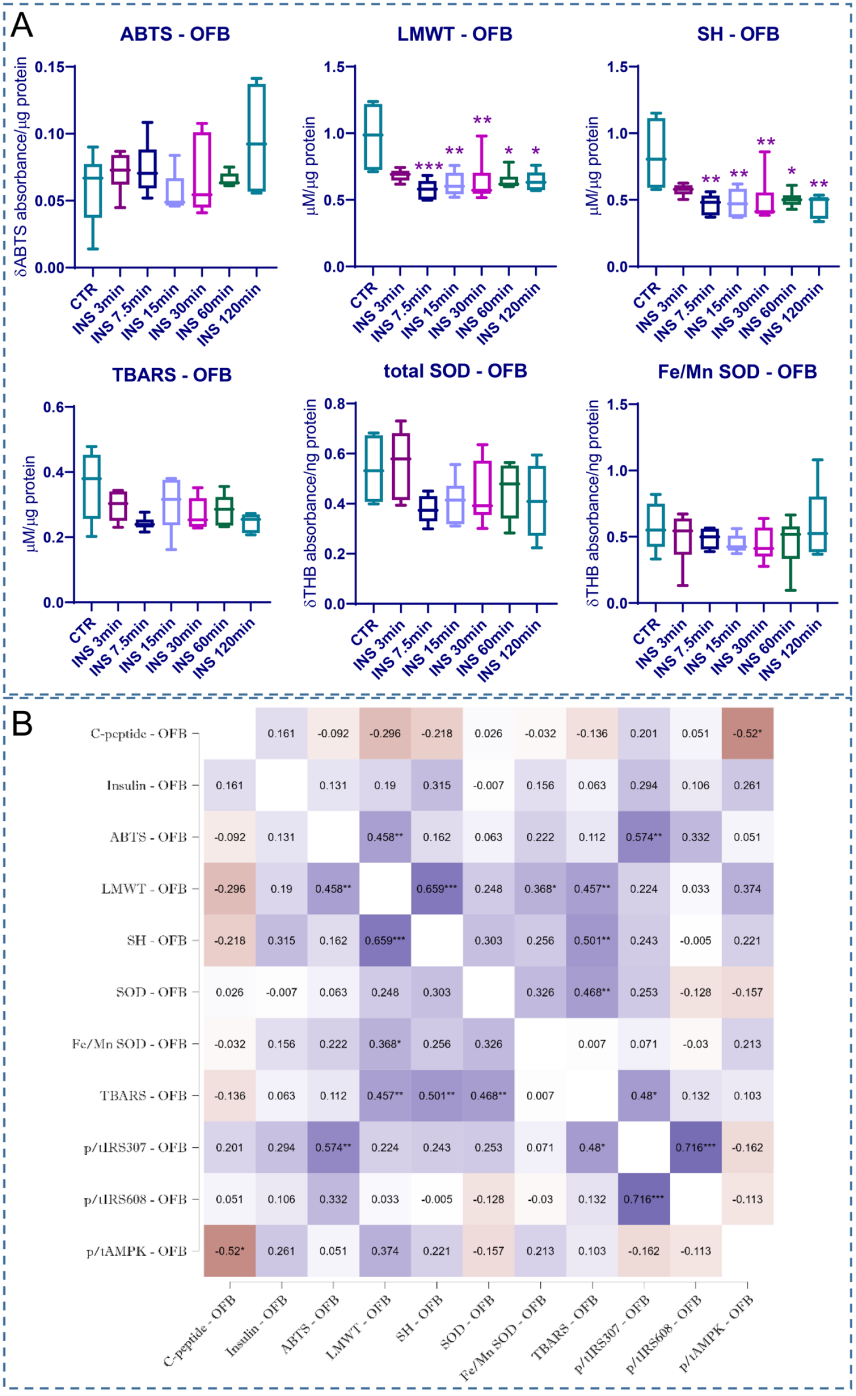
Time‐dependent effect of insulin on redox homeostasis in olfactory bulb. (A) Free thiol group (SH) concentration, low‐molecular‐weight thiol (LMWT) concentration, total reducing capacity (ABTS), superoxide dismutase activity (total SOD), Fe/Mn superoxide dismutase activity (Fe/Mn SOD), and indirect assessment of lipid peroxidation by quantification of thiobarbituric acid reactive substances (TBARS) were determined in olfactory bulb (OFB). Results are presented as box‐and‐whisker plots, and differences between groups were analyzed using Kruskal–Wallis analysis of variance followed by an uncorrected Dunn's test, with the *p* value set at 0.05 (*p < 0.05 vs. control, **p < 0.01 vs. control, ***p < 0.001 vs. control). (B) Redox homeostasis parameters were correlated with insulin and C‐peptide concentrations, as well as with measures of insulin receptor substrate activation (p/tIRS608), insulin receptor substrate inhibition (p/tIRS307), and AMP‐activated kinase activation (p/tAMPK). Spearman's rank correlations are presented as a heatmap, with statistically significant correlations indicated as *p < 0.05, **p < 0.01, ***p < 0.001.

Intranasal insulin administration did not significantly affect total reductive capacity, TBARS levels, or SOD activity in the OFB, but it significantly reduced LMWT and SH concentrations at 7.5 (−40.75%; −44.30%), 15 (−36.63%; −42.96%), 30 (−35.12%; −41.99%), 60 (−34.18%; −39.96%), and 120 (−34.27%; −45.18%) minutes after administration (Figure 6A). A positive correlation was observed between total reductive capacity and p/tIRS307 (ρ = 0.574) (Figure 6B).

#### 3.3.4 Striatum

**FIGURE 7:**
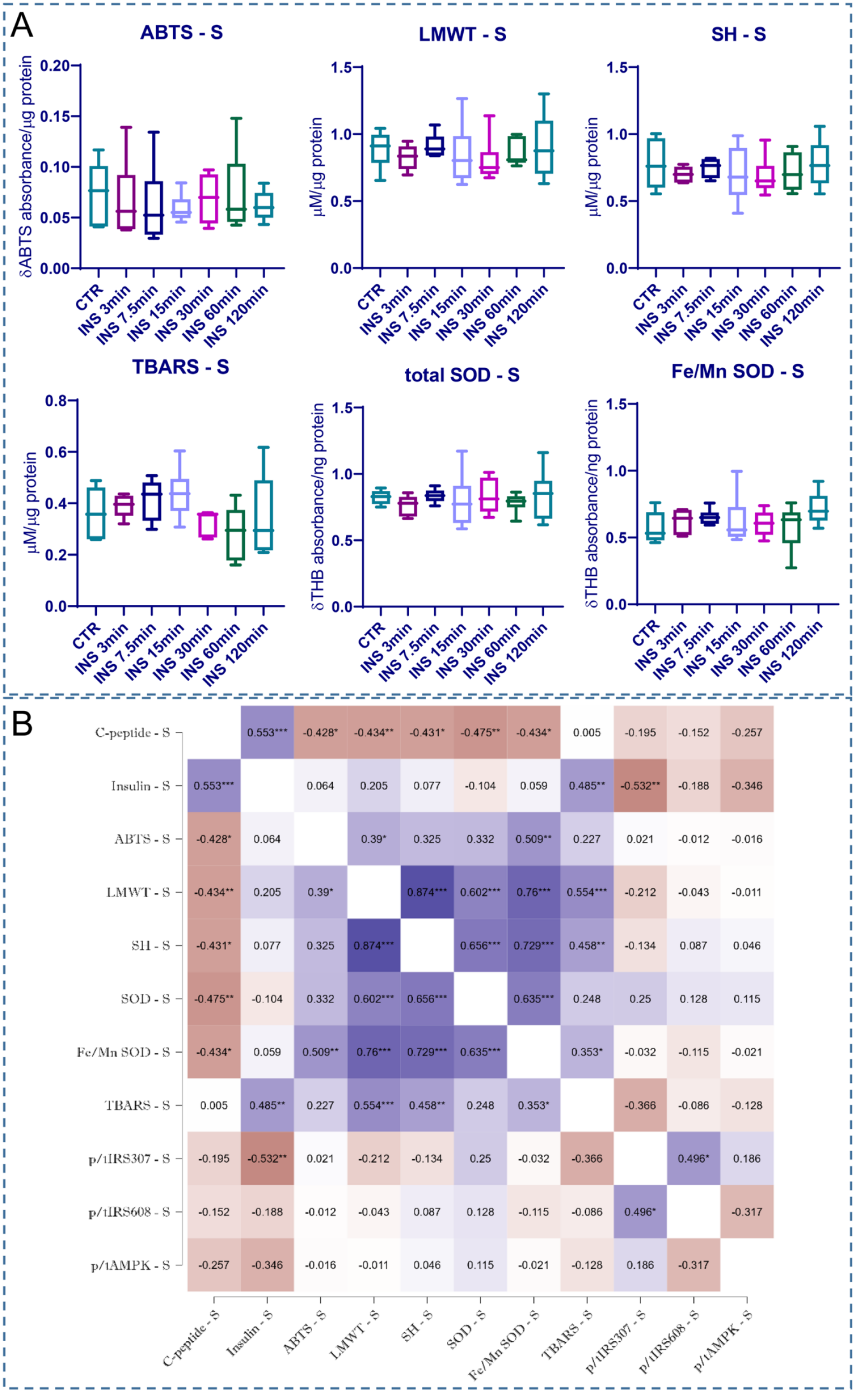
Time‐dependent effect of insulin on redox homeostasis in striatum. (A) Free thiol group (SH) concentration, low‐molecular‐weight thiol (LMWT) concentration, total reducing capacity (ABTS), superoxide dismutase activity (total SOD), Fe/Mn superoxide dismutase activity (Fe/Mn SOD), and indirect assessment of lipid peroxidation by quantification of thiobarbituric acid reactive substances (TBARS) were determined in striatum (S). Results are presented as box‐and‐whisker plots, and differences between groups were analyzed using Kruskal–Wallis analysis of variance followed by an uncorrected Dunn's test, with the *p* value set at 0.05 (*p < 0.05 vs. control, **p < 0.01 vs. control, ***p < 0.001 vs. control). (B) Redox homeostasis parameters were correlated with insulin and C‐peptide concentrations, as well as with measures of insulin receptor substrate activation (p/tIRS608), insulin receptor substrate inhibition (p/tIRS307), and AMP‐activated kinase activation (p/tAMPK). Spearman's rank correlations are presented as a heatmap, with statistically significant correlations indicated as *p < 0.05, **p < 0.01, ***p < 0.001.

Intranasal insulin administration did not significantly affect any measured redox parameter in the striatum (Figure 7A). Weak to moderate correlations (ρ = −0.431 to −0.475) were observed between C‐peptide levels and most oxidative stress parameters, with the exception of TBARS (Figure 7B).

#### 3.3.5 Cerebellum

**FIGURE 8:**
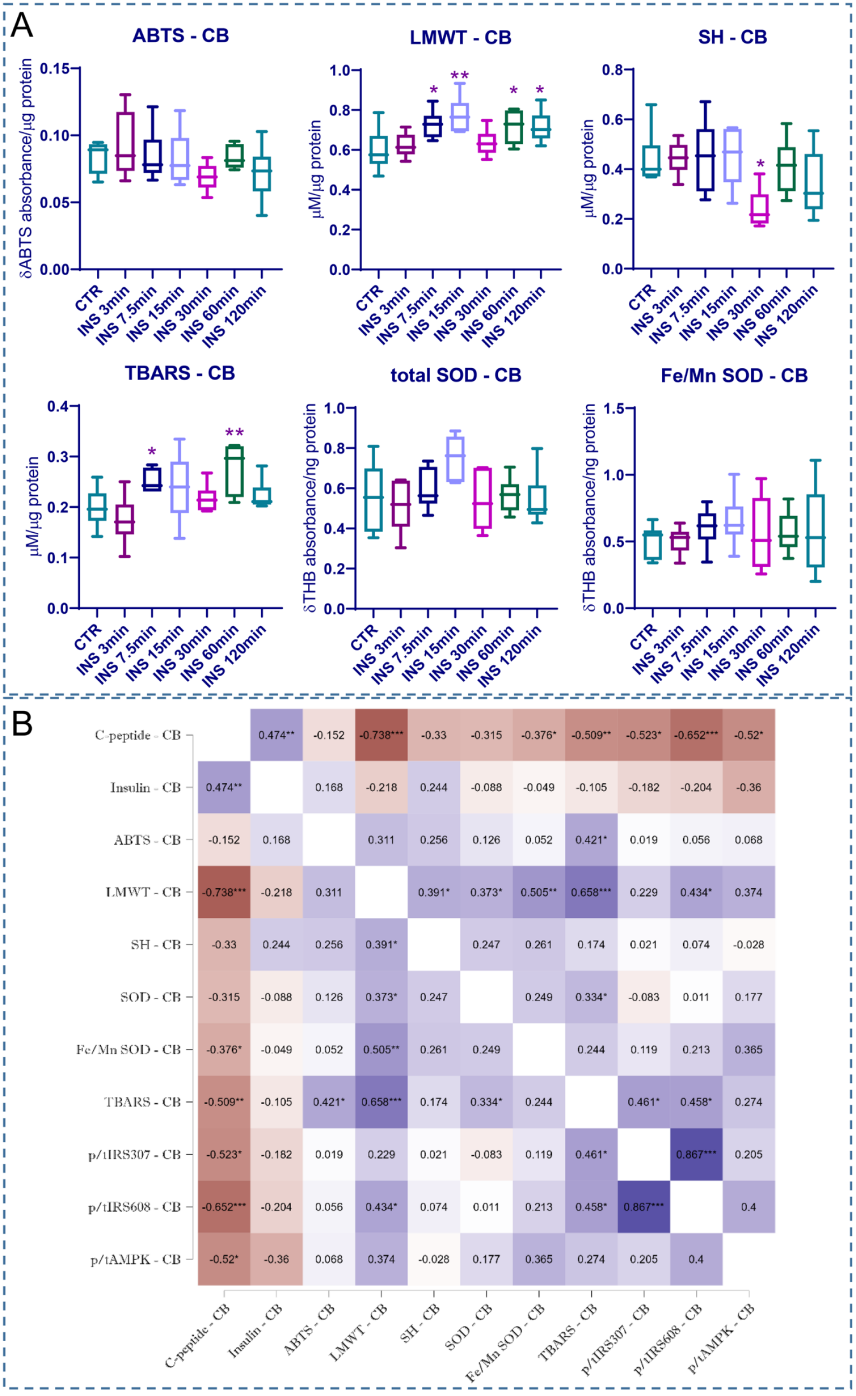
Time‐dependent effect of insulin on redox homeostasis in cerebellum. (A) Free thiol group (SH) concentration, low‐molecular‐weight thiol (LMWT) concentration, total reducing capacity (ABTS), superoxide dismutase activity (total SOD), Fe/Mn superoxide dismutase activity (Fe/Mn SOD), and indirect assessment of lipid peroxidation by quantification of thiobarbituric acid reactive substances (TBARS) were determined in cerebellum (CB). Results are presented as box‐and‐whisker plots, and differences between groups were analyzed using Kruskal–Wallis analysis of variance followed by an uncorrected Dunn's test, with the *p* value set at 0.05 (*p < 0.05 vs. control, **p < 0.01 vs. control, ***p < 0.001 vs. control). (B) Redox homeostasis parameters were correlated with insulin and C‐peptide concentrations, as well as with measures of insulin receptor substrate activation (p/tIRS608), insulin receptor substrate inhibition (p/tIRS307), and AMP‐activated kinase activation (p/tAMPK). Spearman's rank correlations are presented as a heatmap, with statistically significant correlations indicated as *p < 0.05, **p < 0.01, ***p < 0.001.

Total reductive capacity and total SOD and Fe/Mn SOD activities in the cerebellum were not significantly altered following intranasal insulin administration. LMWT concentration was increased at 7.5 (+21.64%), 15 (+29.65%), 60 (+19.85%), and 120 (+19.63%) minutes, while SH concentration was decreased at 30 min (−45.31%) following intranasal administration. In addition, TBARS concentration was increased at 7.5 min (+26.37%) and 60 min (+39.86%) after intranasal insulin administration (Figure 8A). In the cerebellum, a strong (*p* < 0.001) negative correlation was observed between C‐peptide concentration and LMWT levels (ρ = −0.738) and a moderate (*p* < 0.001) negative correlation between C‐peptide and TBARS (ρ = −0.509) (Figure 8B).

#### 3.3.6 Brain Stem

**FIGURE 9:**
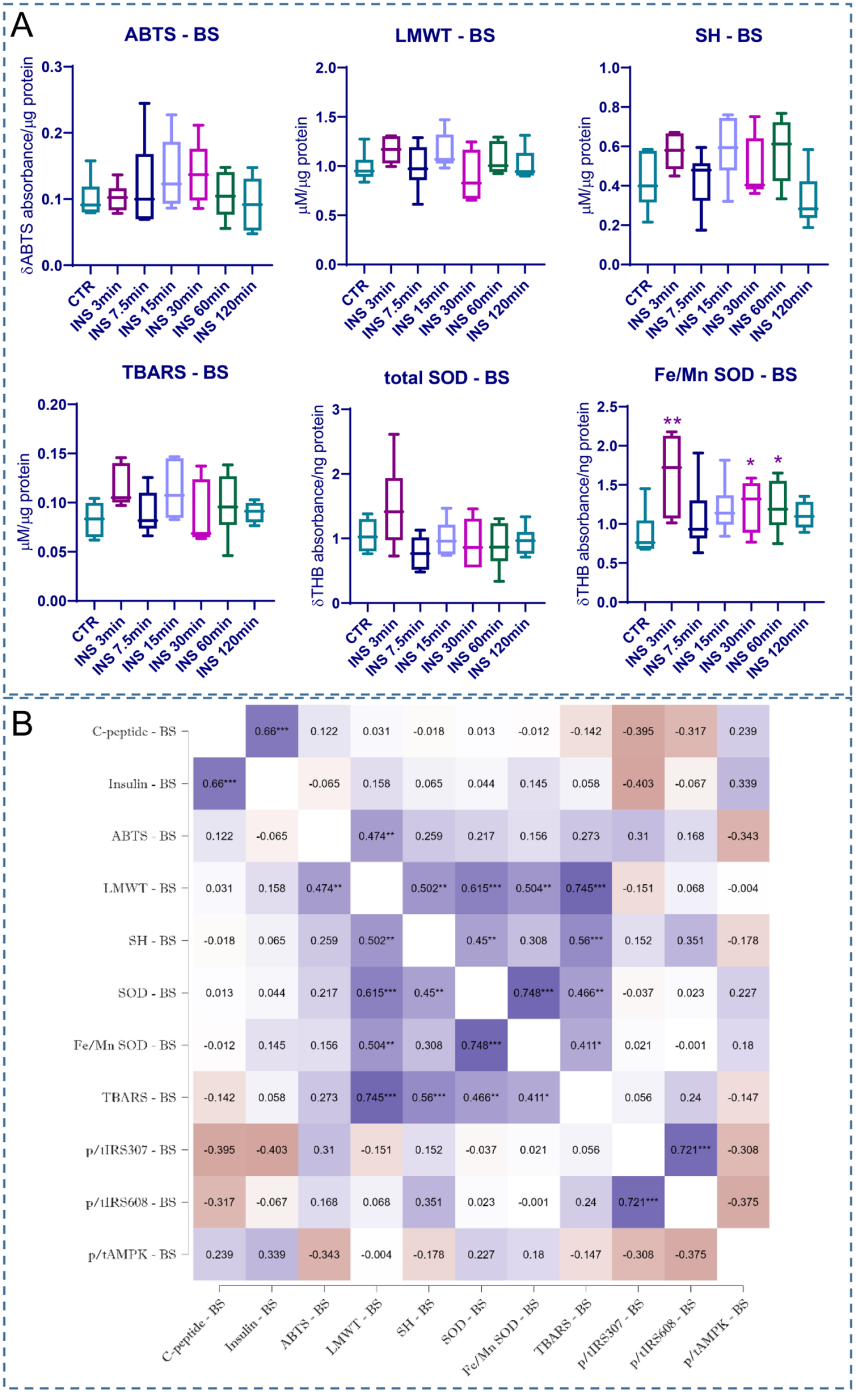
Time‐dependent effect of insulin on redox homeostasis in brain stem. (A) Free thiol group (SH) concentration, low‐molecular‐weight thiol (LMWT) concentration, total reducing capacity (ABTS), superoxide dismutase activity (total SOD), Fe/Mn superoxide dismutase activity (Fe/Mn SOD), and indirect assessment of lipid peroxidation by quantification of thiobarbituric acid reactive substances (TBARS) were determined in brain stem (BS). Results are presented as box‐and‐whisker plots, and differences between groups were analyzed using Kruskal–Wallis analysis of variance followed by an uncorrected Dunn's test, with the *p* value set at 0.05 (*p < 0.05 vs. control, **p < 0.01 vs. control, ***p < 0.001 vs. control). (B) Redox homeostasis parameters were correlated with insulin and C‐peptide concentrations, as well as with measures of insulin receptor substrate activation (p/tIRS608), insulin receptor substrate inhibition (p/tIRS307), and AMP‐activated kinase activation (p/tAMPK). Spearman's rank correlations are presented as a heatmap, with statistically significant correlations indicated as *p < 0.05, **p < 0.01, ***p < 0.001.

Intranasal insulin administration did not significantly change total reductive capacity, LMWT or SH concentrations, TBARS levels, or total SOD activity in the BS. The only significant changes observed were an increase in Fe/Mn SOD activity at 3 (+87.66%), 30 (+41.55%), and 60 (+40.29%) minutes (Figure 9A). In the BS, no significant associations (ρ < 0.5 and *p* > 0.01) were observed between the measured oxidative stress parameters and insulin signaling markers across time after intranasal insulin administration (Figure 9B).

#### 3.3.7 Cerebral Cortices

**FIGURE 10:**
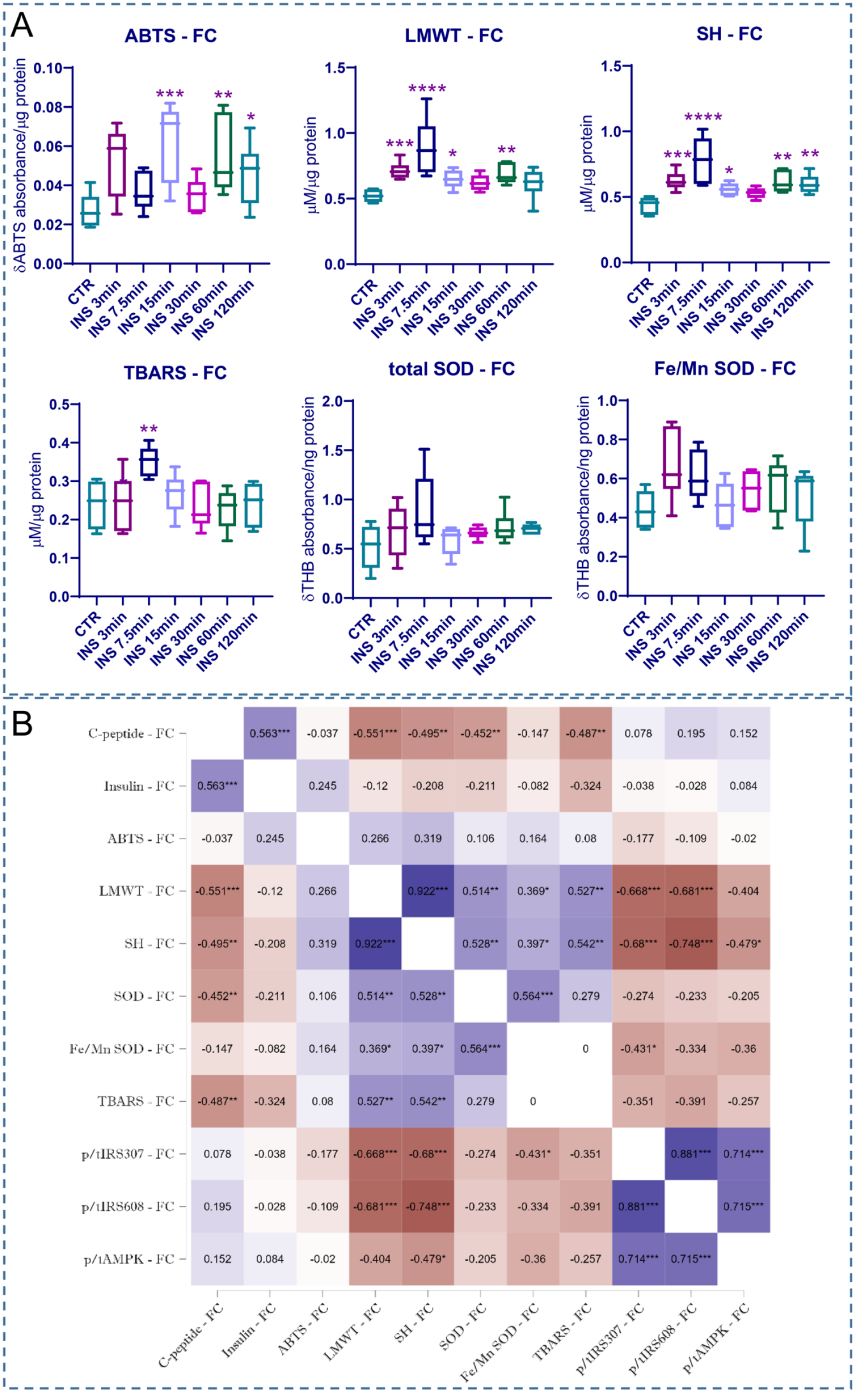
Time‐dependent effect of insulin on redox homeostasis in frontal cortex. (A) Free thiol group (SH) concentration, low‐molecular‐weight thiol (LMWT) concentration, total reducing capacity (ABTS), superoxide dismutase activity (total SOD), Fe/Mn superoxide dismutase activity (Fe/Mn SOD), and indirect assessment of lipid peroxidation by quantification of thiobarbituric acid reactive substances (TBARS) were determined in frontal cortex (FC). Results are presented as box‐and‐whisker plots, and differences between groups were analyzed using Kruskal–Wallis analysis of variance followed by an uncorrected Dunn's test, with the *p* value set at 0.05 (*p < 0.05 vs. control, **p < 0.01 vs. control, ***p < 0.001 vs. control). (B) Redox homeostasis parameters were correlated with insulin and C‐peptide concentrations, as well as with measures of insulin receptor substrate activation (p/tIRS608), insulin receptor substrate inhibition (p/tIRS307), and AMP‐activated kinase activation (p/tAMPK). Spearman's rank correlations are presented as a heatmap, with statistically significant correlations indicated as *p < 0.05, **p < 0.01, ***p < 0.001.

Across cortical regions, redox responses varied by anatomical area. Intranasal insulin administration did not significantly alter total SOD or Fe/Mn SOD activity in the FC. Compared with control, LMWT concentration was increased at 3 (+37.05%), 7.5 (+71.51%), 15 (+24.44%), and 60 (+31.75%) minutes, while SH concentrations were elevated at 3 (+42.33%), 7.5 (+78.95%), 15 (+27.58%), 60 (+40.32%), and 120 (+35.73%) minutes following intranasal administration. TBARS levels were found increased at 7.5 min (+45.7%), whereas total reductive capacity was increased at 15 (+131.96%), 60 (+100.11%), and 120 (+69.22%) minutes after intranasal insulin administration (Figure 10A). In the FC, strong negative correlations (*p* < 0.001) were observed between LMWT concentration and C‐peptide (ρ = −0.551), LMWT and p/tIRS307 (ρ = −0.668), and LMWT and p/tIRS608 (ρ = −0.681). Similarly, SH concentrations showed moderate to strong negative correlations with C‐peptide (ρ = −0.495), p/tIRS307 (ρ = −0.680), and p/tIRS608 (ρ = −0.748) (Figure 10B).

**FIGURE 11:**
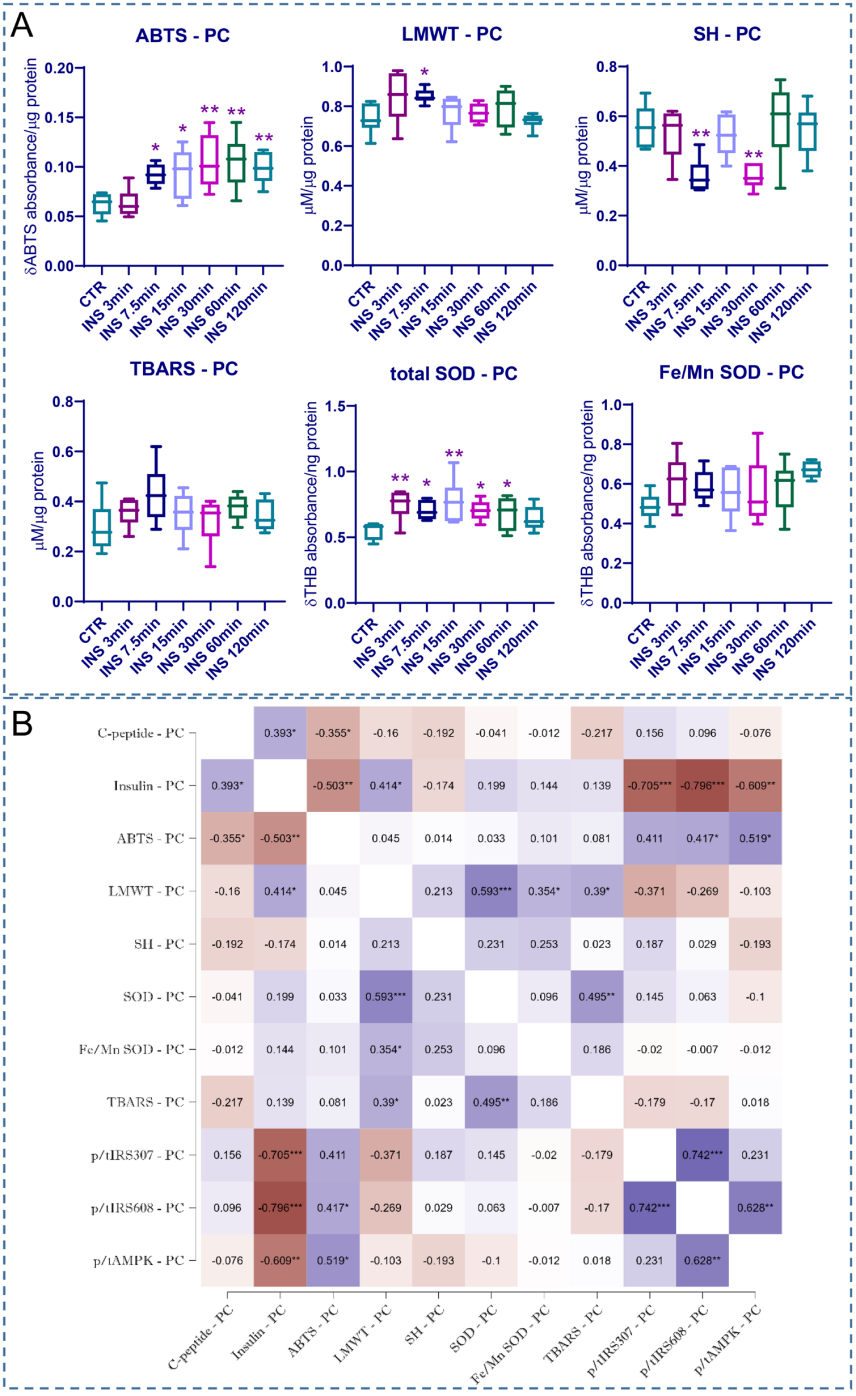
Time‐dependent effect of insulin on redox homeostasis in parietal cortex. (A) Free thiol group (SH) concentration, low‐molecular‐weight thiol (LMWT) concentration, total reducing capacity (ABTS), superoxide dismutase activity (total SOD), Fe/Mn superoxide dismutase activity (Fe/Mn SOD), and indirect assessment of lipid peroxidation by quantification of thiobarbituric acid reactive substances (TBARS) were determined in parietal cortex (PC). Results are presented as box‐and‐whisker plots, and differences between groups were analyzed using Kruskal–Wallis analysis of variance followed by an uncorrected Dunn's test, with the *p* value set at 0.05 (*p < 0.05 vs. control, **p < 0.01 vs. control, ***p < 0.001 vs. control). (B) Redox homeostasis parameters were correlated with insulin and C‐peptide concentrations, as well as with measures of insulin receptor substrate activation (p/tIRS608), insulin receptor substrate inhibition (p/tIRS307), and AMP‐activated kinase activation (p/tAMPK). Spearman's rank correlations are presented as a heatmap, with statistically significant correlations indicated as *p < 0.05, **p < 0.01, ***p < 0.001.

In the PC, intranasally administered insulin increased total reductive capacity from 7.5 to 120 min post‐administration ranging from +47.71% to +68.75% and enhanced total SOD activity from 3 to 60 min ranging from +23.81% to +40.92%. SH concentration was decreased at 7.5 (−35.58%) and 30 (−36.10%) minutes following intranasal administration, whereas LMWT concentration was increased at 7.5 min (+15.32%) (Figure 11A). In the PC, a moderate negative correlation was observed between insulin concentration and total reductive capacity (ρ = −0.503) (Figure 11B).

**FIGURE 12:**
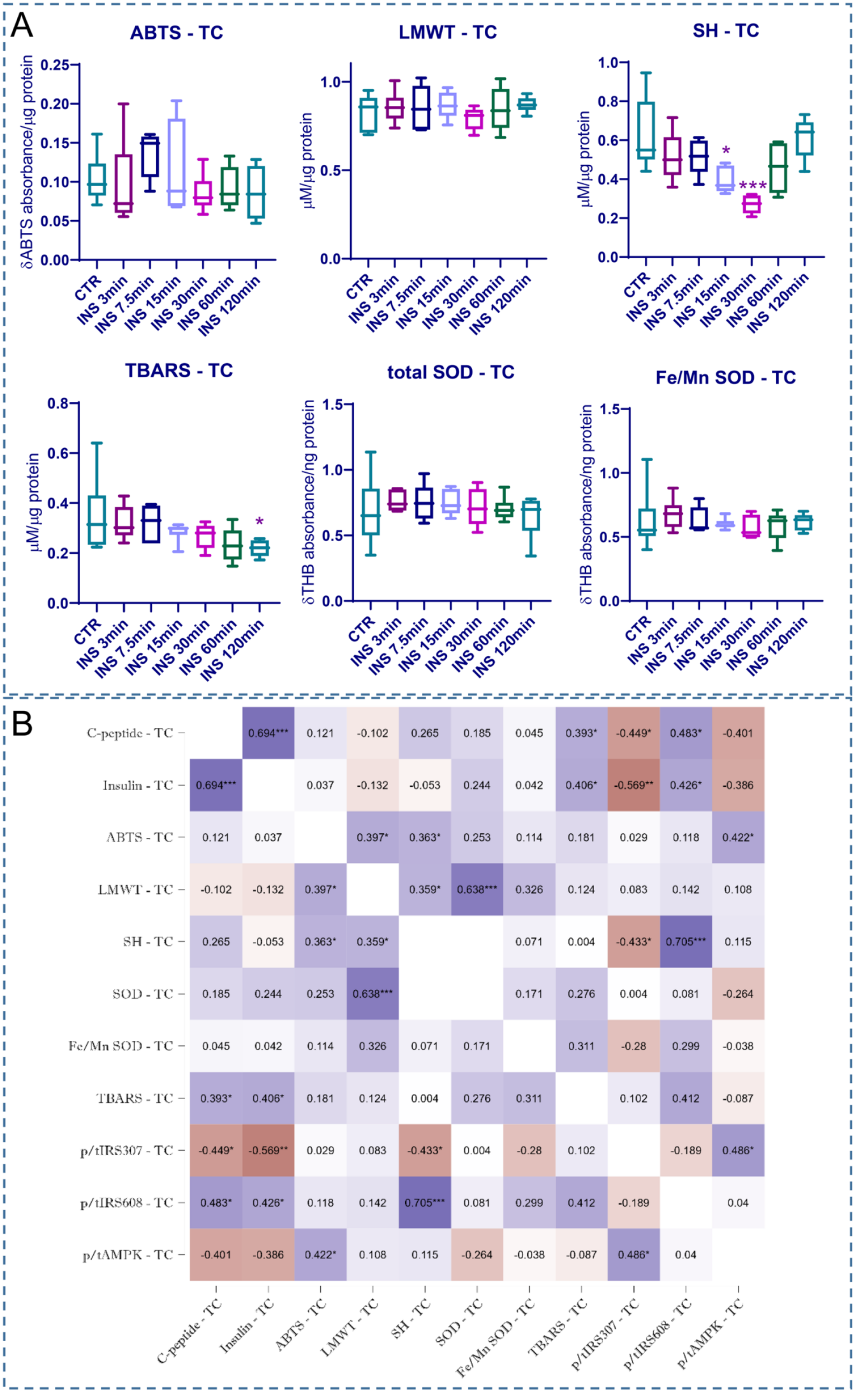
Time‐dependent effect of insulin on redox homeostasis in temporal cortex. (A) Free thiol group (SH) concentration, low‐molecular‐weight thiol (LMWT) concentration, total reducing capacity (ABTS), superoxide dismutase activity (total SOD), Fe/Mn superoxide dismutase activity (Fe/Mn SOD), and indirect assessment of lipid peroxidation by quantification of thiobarbituric acid reactive substances (TBARS) were determined in temporal cortex (TC). Results are presented as box‐and‐whisker plots, and differences between groups were analyzed using Kruskal–Wallis analysis of variance followed by an uncorrected Dunn's test, with the *p* value set at 0.05 (*p < 0.05 vs. control, **p < 0.01 vs. control, ***p < 0.001 vs. control). (B) Redox homeostasis parameters were correlated with insulin and C‐peptide concentrations, as well as with measures of insulin receptor substrate activation (p/tIRS608), insulin receptor substrate inhibition (p/tIRS307), and AMP‐activated kinase activation (p/tAMPK). Spearman's rank correlations are presented as a heatmap, with statistically significant correlations indicated as *p < 0.05, **p < 0.01, ***p < 0.001.

In the TC, SH concentration was found decreased at 15 (−36.64%) and 30 (−56.78%) minutes, while TBARS levels were reduced at 120 (−37.08%) minutes following intranasal insulin administration. Total SOD activtiy, Fe/Mn SOD activity, LMWT concentration, and total reductive capacity were not significantly altered in the TC following intranasal insulin administration (Figure 12A). In the TC, a strong positive correlation (*p* < 0.001) was observed between SH concentration and p/tIRS608 (ρ = +0.705), along with a weaker negative correlation with p/tIRS307 (ρ = −0.433) (Figure 12B).

## 4 Discussion

This study examined the effects of intranasal insulin administration on redox homeostasis in the brain, focusing on different brain regions and epithelia along the entry route. The obtained data complement and clearly align with recently published findings on the pharmacokinetics and pharmacodynamics of intranasally administered insulin [17]. The results of this study show that insulin administration via the nasal mucosa leads to significant, region‐dependent, and time‐specific changes in oxidative stress parameters. The absence of significant systemic redox alterations in plasma supports the concept that intranasal insulin predominantly acts locally within the central nervous system and associated nasal tissues. This observation is consistent with previous reports showing that intranasal insulin bypasses the peripheral circulation and minimizes systemic metabolic effects, including hypoglycemia, while efficiently engaging brain insulin signaling pathways [17, 30]. The localized nature of the redox responses observed here further underscores the suitability of the intranasal route for selectively targeting brain insulin–redox interactions.

The most pronounced redox alterations were observed in the OE and RE, which is expected given that nasal epithelia represent first‐contact regions following intranasal administration [31] and therefore exhibit rapid and robust responses. Notably, acute intranasal insulin induced distinct, region‐specific redox profiles in these tissues, indicating differential activation of insulin‐dependent signaling pathways beginning as early as 7.5 min post‐administration. In the RE, the simultaneous increase in TBARS levels and antioxidant parameters (SOD activity, LMWT and SH concentration, and total reductive capacity) suggests the presence of transient oxidative stress accompanied by compensatory antioxidant upregulation. In contrast, the OE displayed a uniform reduction in both lipid peroxidation and antioxidant parameters, indicating a fundamentally different redox strategy. These opposing redox responses likely reflect fundamental structural and functional differences between the RE and the OE (Table 1). The RE consists primarily of ciliated and non‐ciliated columnar epithelial cells, goblet cells, basal progenitors, and submucosal glands with strong trigeminal innervation, whereas the OE is a specialized neuroepithelium responsible for olfactory transduction and contains olfactory sensory neurons (OSNs), sustentacular cells, Bowman's glands, microvillous and tuft cells, and basal stem cells. These differences also affect brain transport: molecules absorbed through the OE can rapidly access the CNS via OSN perineural pathways, while transport through RE‐associated trigeminal pathways is considerably slower [32]. The OE expresses insulin signaling components [33], and exhibits high insulin‐binding capacity and marked insulin responsiveness. Experimental studies demonstrated protective effects of insulin in the OE by suppressing apoptosis [34] and promoting cell recovery following injury [34]. Barrier and immune properties further distinguish these epithelia. The OE forms a selective blood–olfactory barrier and lacks mucociliary clearance, resulting in delayed and reactive immune responses, whereas the RE possesses robust innate defenses, including mucociliary clearance, antimicrobial peptides, abundant immune cells, and high vascularization [32]. In addition, the OE demonstrates greater P‐glycoprotein (P‐gp)‐mediated efflux activity than the RE [35]. Although insulin is not known to be a P‐gp substrate, insulin signaling helps maintain normal BBB P‐gp expression and function [36]. IR activation can enhance mitochondrial metabolism [37] and transiently increase ROS, which in turn may trigger Nrf2‐mediated antioxidant defenses [38], whereas excessive or prolonged ROS can promote maladaptive stress responses [38]. Within this framework, the RE response, characterized by increased TBARS with elevated antioxidant capacity, likely represents a coordinated oxidative–antioxidative adaptation rather than overt oxidative damage, whereas the OE showed parallel reductions in lipid peroxidation and antioxidant markers, suggesting a different redox strategy that may reflect its neuronal composition and lower antioxidant reserve. Although insulin can activate antioxidant responses, the decrease in antioxidant markers may also reflect depletion of redox defenses. This may be related to the intrinsic vulnerability of neurons to lipid peroxidation and oxidative damage due to their relatively weak antioxidant defenses [39] and their high metabolic demand [40]. Overall, the distinct cellular and barrier properties of the OE and RE highlight the importance of epithelial context in intranasal insulin responses.

**TABLE 1:** Epithelia‐ and brain tissue‐specific effects of intranasal insulin on redox homeostasis. The table summarizes the main direction of change and the affected oxidative stress‐related parameters.

Tissue	Direction of change	Most affected parameter(s)
RE	**↑**	Total reductive capacity, LMWT, SH, TBARS, total SOD and FeMn SOD
OE	**↓**	Total reductive capacity, LMWT, SH, TBARS, total SOD and FeMn SOD
HPC	**↓**	Total reductive capacity and LMWT
HPT	**↓**	LMWT
OFB	**↓**	LMWT and SH
TC	**↓**	SH and TBARS
S	X	No change
CB	**↑**	LMWT and TBARS
BS	**↑**	FeMn SOD
FC	**↑**	Total reductive capacity, LMWT, SH and TBARS
PC	**↑**	Total reductive capacity, LMWT and total SOD

*Note:* Free thiol group (SH) concentration.

Abbreviations: BS, brain stem; CB, cerebellum; FC, frontal coritices; Fe/Mn SOD, Fe/Mn superoxide dismutase activity; HPC, hippocampus; HPT, hypothalamus; LMWT, low‐molecular‐weight thiol concentration; OE, olfactory epithelia; OFB, olfactory bulb; PC, parietal coritices; RE, respiratory epithelia; S, striatum; TBRAS, lipid peroxidation by quantification of thiobarbituric acid reactive substances; TC, temporal; Total SOD, superoxide dismutase activity.

In epithelial regions, a positive correlation was observed between AMPK activation and thiol parameters and SOD activity, suggesting a potential role in regulating local redox balance following intranasal insulin administration. AMPK is a redox‐sensitive metabolic sensor activated during metabolic or oxidative stress [41], that promotes antioxidant defense by reducing mitochondrial and NADPH oxidase‐derived ROS and by promoting the expression of antioxidant genes [42], preserving NADPH availability, and enhancing glutathione‐dependent buffering [43]. These mechanisms are consistent with the observed association between AMPK activity and thiol‐based antioxidant markers. AMPK activity, however, is context dependent: moderate activation supports recovery from metabolic stress, whereas prolonged activation may promote apoptosis [44]. In neuronal systems, oxidative stress activates AMPK, while insulin signaling suppresses this response, restoring mitochondrial function and normalizing apoptotic markers [45, 46]. Balanced regulation between insulin signaling and AMPK is also essential for mitochondrial quality control, while its disruption during insulin resistance may increase vulnerability to oxidative damage [46]. AMPK‐mediated antioxidant regulation in the CNS also appears largely astrocyte‐specific [47], which may explain why AMPK‐associated antioxidant responses were evident in epithelial tissues but not in the examined brain regions. Together, these findings suggest that intranasal insulin may indirectly enhance epithelial antioxidant protection through AMPK‐associated restoration of thiol redox balance. Increased local insulin exposure may trigger feedback inhibition of insulin signaling, reflected by elevated p/tIRS307 phosphorylation, thereby secondarily promoting AMPK activation [17]. However, because the present observations are correlative, further mechanistic studies are needed to confirm this relationship.

Interestingly, IRS inhibition negatively correlated with free thiol (SH) levels in the OE, TC, and FC, indicating that greater IRS inhibition was associated with lower thiol availability. Because IRS‐mediated insulin signaling supports PI3K/Akt‐dependent glucose uptake, mitochondrial metabolism, and antioxidant defense, inhibitory IRS phosphorylation may impair cellular redox homeostasis. As we have mentioned, acute mitochondrial activation can transiently increase ROS production and activate Nrf2‐dependent antioxidant pathways [48]. As thiol groups represent a major component of the cellular redox buffer, increased oxidative burden can lead to their oxidation and depletion. In this context, the observed decrease in SH groups may reflect enhanced ROS‐mediated consumption of thiol pools and induction of antioxidant response, since transcription and translation processes take a longer time. In the TC, where insulin induced early IRS activation followed by later inhibitory phosphorylation [17], IRS activity positively correlated with SH levels, supporting a role for insulin signaling in redox regulation. This pattern may reflect an initial insulin‐driven enhancement of antioxidant defenses and/or increased mitochondrial ROS production that subsequently triggers feedback inhibition of IRS signaling. Indeed, mitochondrial superoxide production can trigger inhibitory IRS‐1 Ser307 phosphorylation and IRS proteolysis, attenuating insulin signaling that limits further mitochondrial substrate flux and ROS generation, thereby functioning as part of a cellular antioxidant defense mechanism aimed at restoring energetic balance [49, 50]. In this context, insulin resistance may function alongside AMPK as a stress‐adaptive mechanism protecting cells from metabolic overload. The examined brain regions also showed marked heterogeneity in the magnitude and timing of redox responses to intranasal insulin, likely reflecting regional differences in metabolic demand, antioxidant capacity, insulin receptor density, cell composition, and intracellular signaling architecture.

The most striking finding was the negative correlation between C‐peptide concentrations and oxidative stress parameters (particularly LMWT) across several brain regions and all examined epithelial tissues (RE, OE, HPT, FC, S, and CB). In our previous study using the same samples, intranasal insulin increased C‐peptide levels in plasma and several brain regions immediately after administration, followed by a decrement in certain brain regions [17]. Because plasma C‐peptide also increased, these regional brain changes may reflect peripheral secretion and BBB transport of C‐peptide, although local CNS production cannot be excluded [17]. Similar associations between C‐peptide and redox parameters have been reported previously. Konukoğlu et al. observed that erythrocyte glutathione levels negatively correlated with C‐peptide during the oral glucose tolerance test [51]. Although once considered only a by‐product of insulin biosynthesis, C‐peptide is now recognized to exert important physiological effects [52], including reduction of mitochondrial superoxide production [53], activation of AMPKα signaling which suppresses intracellular and mitochondrial ROS production, preserves mitochondrial integrity, and prevents apoptosis [54], and suppression of NAD(P)H oxidase activity [54]. Because NADPH is needed for glutathione reductase to regenerate reduced glutathione (GSH) from its oxidized form, these mechanisms may indirectly support thiol‐based antioxidant buffering and cellular redox balance. Consistently, experimental studies show that C‐peptide reduces oxidative stress–related β‐cell [55], reduces neuronal damage, and improves cognitive outcomes in diabetes models [56]. Taken together, these findings suggest that C‐peptide may participate in cellular antioxidant defense mechanisms, potentially through modulation of mitochondrial ROS production and AMPK‐dependent stress responses. The negative correlation between C‐peptide and thiol‐based antioxidant parameters may therefore reflect a compensatory response to oxidative challenge, although its precise role in brain redox regulation remains unclear.

The absence of a simple linear relationship between insulin signaling and oxidative stress markers across most regions indicates that insulin exerts bidirectional and time‐dependent effects on CNS redox homeostasis (Table 1). These findings highlight the importance of considering regional heterogeneity, temporal dynamics, and the role of nasal epithelia as integral components of insulin transport and signaling. Consequently, studies investigating intranasal insulin should account not only for dose and frequency of administration, but also for the timing of measurements and the specific brain regions analyzed. Several limitations should be acknowledged. Although multiple brain regions and epithelial tissues were examined, the analysis did not include several important redox‐regulating systems, such as Nrf2 signaling or glutathione peroxidase. In addition, the use of an animal model limits direct extrapolation to human physiology, particularly with respect to dose, pharmacokinetics, and clinical conditions. Finally, while associations between insulin signaling and redox parameters were identified, causal relationships cannot be definitively established and the effect of chronic insulin administration could differ from acute response. In conclusion, the present study demonstrates that intranasal insulin modulates CNS redox homeostasis in a highly region‐ and time‐dependent manner. These findings support the growing recognition of insulin as an important neuromodulatory molecule involved not only in metabolic regulation but also in oxidative balance and neuronal protection. Understanding the regional and temporal complexity of insulin‐mediated redox regulation may therefore be essential for optimizing intranasal insulin–based therapeutic strategies for neurological and neurodegenerative disorders.

## Author Contributions

A. Knezovic, J. Osmanovic Barilar, and M. Salkovic‐Petrisic designed research; L. Vlahov, J. Osmanovic Barilar, A. Krsnik, L. Mihalic, A. Babic Perhoc, D. Virag, J. Homolak, and A. Knezovic performed research; A. Knezovic, L. Vlahov, and J. Osmanovic Barilar analyzed data; all authors were involved in drafting and revising the manuscript.

## Funding

This work was supported by the Croatian Science Foundation under the project number HRZZ‐2022‐10‐1895, DOK‐NPOO‐2023‐10‐3354, and DOK‐2025‐02‐5472 and University of Zagreb project (10106‐24‐1453). The work of doctoral students Antonia Krsnik and Luka Mihalic has been fully supported by the “Young researchers' career development project—training of doctoral students” of the Croatian Science Foundation.

## Conflicts of Interest

The authors declare no conflicts of interest.

## Data Availability

The datasets generated and/or analyzed during the current study are available in the Mendeley Data repository: Knezovic, Ana (2026), “Time‐ and region‐specific effects of intranasal insulin on oxidative stress parameters in the rat brain”, Mendeley Data, V1, doi:https://doi.org/10.17632/ptfybnhp5g.1
